# The Temporal Structure of Behaviour and Sleep Homeostasis

**DOI:** 10.1371/journal.pone.0050677

**Published:** 2012-12-05

**Authors:** Vladyslav V. Vyazovskiy, Irene Tobler

**Affiliations:** 1 Institute of Pharmacology and Toxicology, University of Zurich, Zurich, Switzerland; 2 Department of Biochemistry and Physiology, University of Surrey, Guildford, Surrey, United Kingdom; Imperial College London, United Kingdom

## Abstract

The amount and architecture of vigilance states are governed by two distinct processes, which occur at different time scales. The first, a slow one, is related to a wake/sleep dependent homeostatic Process S, which occurs on a time scale of hours, and is reflected in the dynamics of NREM sleep EEG slow-wave activity. The second, a fast one, is manifested in a regular alternation of two sleep states – NREM and REM sleep, which occur, in rodents, on a time scale of ∼5–10 minutes. Neither the mechanisms underlying the time constants of these two processes – the slow one and the fast one, nor their functional significance are understood. Notably, both processes are primarily apparent during sleep, while their potential manifestation during wakefulness is obscured by ongoing behaviour. Here, we find, in mice provided with running wheels, that the two sleep processes become clearly apparent also during waking at the level of behavior and brain activity. Specifically, the slow process was manifested in the total duration of waking periods starting from dark onset, while the fast process was apparent in a regular occurrence of running bouts during the waking periods. The dynamics of both processes were stable within individual animals, but showed large interindividual variability. Importantly, the two processes were not independent: the periodic structure of waking behaviour (fast process) appeared to be a strong predictor of the capacity to sustain continuous wakefulness (slow process). The data indicate that the temporal organization of vigilance states on both the fast and the slow time scales may arise from a common neurophysiologic mechanism.

## Introduction

While it is established that staying awake leads to an enhanced intensity of subsequent sleep [1], measured as an increase in NREM sleep EEG slow wave-activity (0.5–4 Hz, SWA), it is not yet known which specific processes occurring during waking are responsible for sleep need [2]. Recent studies suggest that the increase of sleep propensity is determined by the duration of wakefulness and by waking activities [3], [4]. On the other hand, local changes in sleep SWA are brought about by a sensory load [5] or synaptic plastic processes [6]. The mechanisms underlying the sleep-wake-dependent changes in brain activity are still unresolved. There are several candidates believed to be implicated in sleep need: regulation of brain metabolism [7], [8], activity-dependent release of cytokines [9], [10], and synaptic plasticity [11], [12]. Surprisingly, it is still unknown what triggers the transition from waking to sleep, once sleep propensity reaches a level that can no longer be sustained.

Under physiological conditions, spontaneous behavioral states do not persist for longer than minutes or hours, tending to change frequently. The probability with which an animal enters a particular state is not always the same. The preceding history of sleep, waking and activity, circadian factors, external stimuli, and many other processes govern the changes in behaviour and brain state. The spontaneous dynamics of vigilance states can be roughly described by two distinct temporal scales. The first process, occurring on a relatively fast time scale ranging from minutes in small rodents to 1–2 hours in humans and large mammals, is manifested in a regular alternation between episodes of NREM and REM sleep [13], [14], [15], [16], or in the frequency of sleep-wake transitions [17]. NREM sleep episodes are usually longer and are often followed by a shorter episode of REM sleep. The NREM – REM sleep cycle repeats itself several times before arousal [18]. Empirical and modeling studies suggest that the NREM-REM sleep cycle arises from a mutual inhibition of two distinct interconnected populations of neurons in the brainstem and in the hypothalamus [19], [20]. The propensity for REM sleep builds up during NREM sleep [21] precipitating its termination, or during both wake and NREM sleep [22], [23], [24].

The second process, occurring on a slower time scale of several hours or days, reflects the time course of a sleep-wake-dependent homeostatic Process S that regulates the amount of waking and sleep throughout 24 h, as well as NREM sleep intensity, measured as EEG slow-wave activity (EEG power in the 0.5–4.0 Hz band) [1], [13]. Modeling studies revealed that sleep pressure increases during waking and dissipates during sleep according to exponential functions with specific time constants [25], [26], [27], [28]. Notably, the time constant of the increase (Ti) of Process S is also usually larger than the corresponding time constant of its decrease (Td) in both humans and rodents [13], [27], [29]. Likewise, the daily amount of waking is usually larger than the amount of sleep in both humans [30] and rodents [31]. The neurophysiologic mechanisms that account for the daily amount of waking and sleep and for their temporal dynamics are still unknown.

While the slow homeostatic process seems to fulfill an important recovery function [2], [14], neither the underlying mechanisms, nor its relationship to the periodicity in sleep structure is clear. The inherent difficulty in bridging the gap between the fast and the slow process that shape the temporal dynamics of vigilance states arises from the fact that their manifestation in waking is obscured. Specifically, while homeostatic sleep propensity increases during waking, its main marker, EEG slow wave activity, is apparent during NREM sleep. Several studies have identified a waking counterpart of both the slow homeostatic process and the faster periodicity. Specifically, EEG power within slower EEG or local field potential (LFP) frequencies, as well as patterns of cortical neuronal activity in awake animals, change systematically as a function of preceding waking duration [4], [32], [33], [34]. An ultradian periodicity in waking behaviour was reported for several species [35], [36], [37]. It is, however, a formidable task to separate state changes triggered by intrinsic vs. extrinsic influences, since the waking state is induced both by the brain and, more often, by sensory inputs from the environment and affected by the ongoing behavior of the animal. Even more importantly, it remains unclear whether the two processes – the slow and the fast one - are related to each other.

In this study, we made use of the spontaneous tendency of mice to run in a running wheel (RW) to investigate the time structure of waking behavior, both on a slow and a fast time scale. The RW-activity monitoring was complemented by EEG and EMG recordings to determine vigilance states. While the mice stayed awake continuously for at least several hours, a clear periodicity within waking was manifested by running bouts that alternated with periods of relative inactivity, during which the mice were always awake, as evident by EEG and EMG activity. The periodicity in waking showed a remarkable similarity to the periodic structure in sleep, manifested in a NREM-REM sleep cycle. Notably, the periodic structure in waking and sleep appeared to be related to the 24-h amount of waking and sleep, as well as to the capacity to sustain prolonged wakefulness. Our data suggest that the temporal organization of wakefulness and sleep on a fast time scale is an integral part of the slow process of sleep homeostasis.

## Results

### Running Wheel Activity and Sleep: Intra Individual Stability and Inter Individual Variability

**Figure 1:**
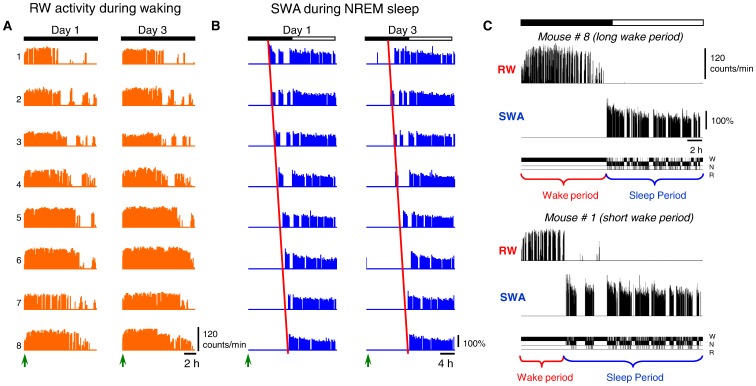
Daily profile of waking and sleep. Wheel running (RW counts/min) and NREM sleep SWA (SWA, slow wave activity, EEG power between 0.5–4 Hz as percentage of 24-h mean in NREM sleep) of the n = 8 individual mice on Days 1 and 3. The animals are ordered from mouse 1 to 8 according to increasing amount of wheel running on Day 1 (**A**). This order of individuals 1–8 resulted in the same order for day 3 and remarkably, in the same ascending order of increasing sleep latency after dark onset on day 1 and 3 (**B**; dark onset marked by arrows), determined by appearance of SWA in NREM sleep. Note the remarkable intra individual stability but inter individual variability of the global pattern of RW-activity and waking duration. (**C**) The 24-h profiles of wheel running, NREM sleep SWA and corresponding hypnograms of two individual mice at two extreme ends of the distribution of spontaneous waking duration (the light-dark cycle is marked by the white and black bar at the top).

Providing mice with a running wheel revealed for how long they could sustain continuous spontaneous waking. Specifically, we hypothesized that mice would desist from running for longer than just a brief period of time only when sleep pressure attains a level that they could no longer tolerate. During the light period, the mice ran very rarely, whereas at dark onset, they ran in the wheel for many hours before engaging in a consolidated sleep period (Fig. 1A). Individual mice differed considerably in the total duration of this waking period, differing by more than 5 hours (Fig. 1A,C). Specifically, after dark onset, individual mice were continuously awake for 355–677 minutes (mean 526.5±43.6 min, SEM) on Day 1 and for 404–741 min (mean 569.0±47.5 min) on Day 3. This difference was reflected in highly variable sleep latency (Fig. 1B), suggesting that it might reflect differential rates of accumulation of homeostatic sleep pressure between individuals. Indeed, despite the pronounced differences in the duration of the waking period from the dark onset until the first consolidated sleep period, the initial levels of NREM SWA during the first hour in subsequent sleep were remarkably similar. Specifically, on Day 1, when we divided the 8 mice into two sub-groups of n = 4, based on long and short waking (629.0±11.95 and 424.0±18.05 min respectively), the corresponding values of SWA (as % of mean NREM sleep SWA over all three days) were 143.3±2.6% and 143.6±2.3%. Consistently, on Day 3, despite almost 4 hours difference in waking duration between long-wake and short-wake subgroups (688.5±13.6 vs. 449.5±8.1 min), the values of SWA were again practically identical (147.8±0.9 vs, 147.2±1.9). These individual differences in the capacity to sustain prolonged wakefulness provide a unique opportunity to gain insights into the underlying mechanisms [38], [39].

**Figure 2:**
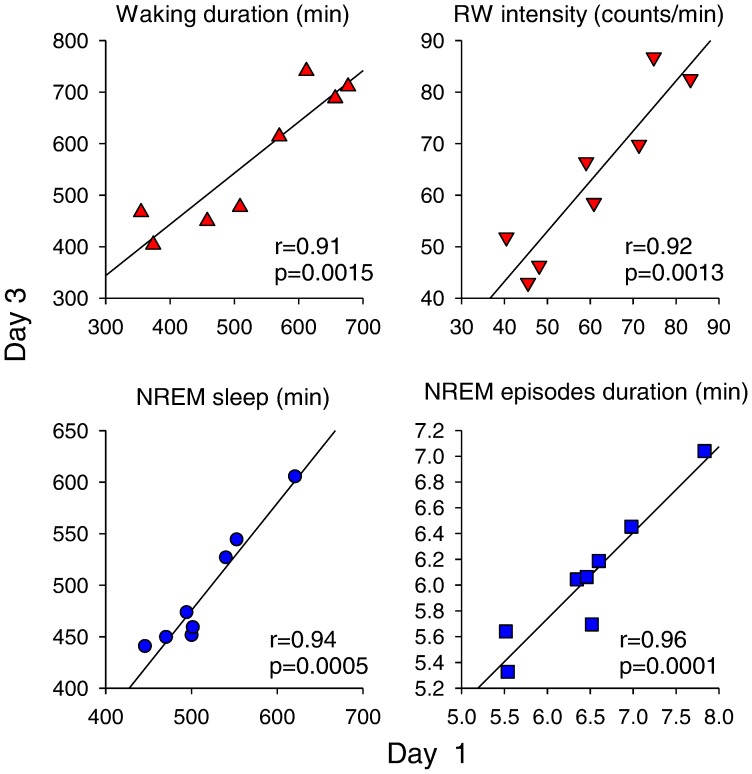
Intra individual stability and inter individual variability of waking and sleep. Correlation of four variables between Day 1 and 3: Waking duration starting from dark onset until the occurrence of the first consolidated sleep period, running wheel intensity (RW counts/min within Run bouts), total 24-h NREM sleep amount and the mean duration of NREM sleep episodes (n = 8 mice). r- and p-values correspond to Pearson correlations between the variables. Note the remarkable intra individual stability of all four variables between the two days.

It should be noted that the duration of waking (latency to consolidated sleep), as well as running wheel intensity within individual mice, were highly consistent from Day 1 to Day 3 (p-values >0.9; Pearson correlation; Fig. 2). The intra individual stability was manifested also in the 24-h amount of NREM sleep, as well as in the average duration of NREM sleep episodes (Fig. 2). Since all the animals were kept undisturbed in identical standardized light-dark conditions, the stability of spontaneous temporal dynamics of waking and sleep suggests that it is likely governed by an intrinsic neurophysiologic mechanism.

### Similar Temporal Organization within Waking and Sleep

**Figure 3:**
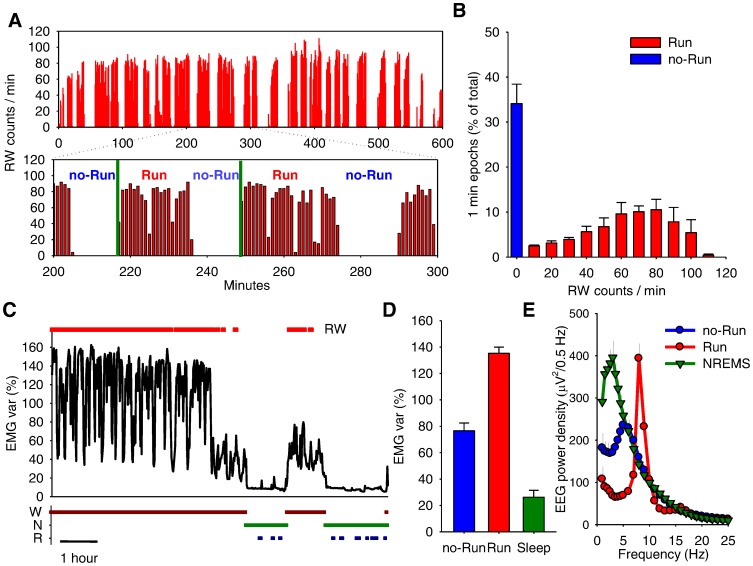
Temporal structure of running wheel activity. (**A**) Wheel running profile (RW) of an individual mouse depicting a running period (600 min; based on one-minute resolution) and an enlarged representative 100-min interval (between minute 200–300) illustrating the temporal structure of RW-activity. Note that running occurs in distinct consolidated bouts (Run) interrupted by the absence of running (no-Run), during which the mouse remains awake according to vigilance state scoring. The two vertical green lines depict a typical Run – no-Run cycle. (**B**) Distribution of all 1-min epochs during the running period (defined as the time interval beginning at dark onset until the first consolidated sleep period), as a function of RW-counts. Note the bimodal distribution, with a distinct peak at 0 counts/min and a second, broader peak between 60–80 counts/min. Mean values +SEM, n = 8 (Days 1 and 3 averaged per mouse). (**C**) Time course of EMG variance in a representative individual during a 9-hour interval encompassing the period with RW-activity (denoted by the bar above the curve) and subsequent sleep. The corresponding vigilance states, waking, NREM and REM sleep (W, N and R) determined by 4-sec EEG and EMG scoring are shown at the bottom. (**D**) EMG variance (mean +SEM) represented as percentage of the mean over the 3 days, shown for waking epochs without running (no-Run), with RW-activity (Run), and sleep (based on scoring of EEG and EMG). (**E**) EEG power density in waking during Run-bouts, no-Run bouts and NREM sleep on Day 1. Curves connect mean values (n = 8) of absolute EEG power density in the frontal derivation. Values are plotted at the upper limit of each frequency bin.

While previous studies using RW-activity addressed the total amount of running, its distribution, or average running-wheel intensity, the fine architecture of RW-activity remained unexplored. We hypothesized that investigating the time structure of RW-activity might provide important insights into the processes underlying the temporal organization of behavior in general. Close inspection of the individual records revealed that each mouse alternated between two distinct stages: running bouts when the mouse ran in the wheel continuously (Run bouts), followed by intermediate periods when the mouse was relatively immobile (no-Run bouts). Fig. 3A illustrates a typical example of this behaviour in an individual mouse. Plotting the distribution of the 1-min epochs for the time interval between dark onset and the occurrence of the first consolidated sleep episode as a function of the corresponding RW-counts revealed a clear bimodality with a distinct peak at 0 counts/min and a second, broader peak between 60–80 counts/min (Fig. 3A).

To investigate the possibility that the mice were asleep during the intermediate no-Run bouts, we performed several complementary analyses. Fig. 3C illustrates the time course of EMG variance during a period of waking with RW-activity followed by sleep. Note that during waking, the EMG drops at regular intervals, corresponding to no-Run bouts, but never reaches the levels typical for sleep. On average, the EMG activity during no-Run bouts was about three times higher as compared to the 24-h average in sleep (p = 7.7185e-007, paired t-test, Fig. 3D). Consistently, IR-activity during no-Run bouts, while substantially lower than during Run-bouts, was about 10 times above the corresponding values in sleep (no-Run: 4.1±0.5, Run: 19.2±5.6, sleep: 0.3119±0.1 counts/min; no-Run vs. sleep: p = 3.1651e-004, paired t-test). Finally, computing the EEG power spectra showed that during no-Run bouts, brain activity was distinctly different from sleep (e.g. SWA decreased by ∼ 50%, p = 0.0011, paired t-test). In addition, during no-Run bouts, fast theta activity (∼9 Hz) was virtually absent, while slower EEG activities predominated.

**Figure 4:**
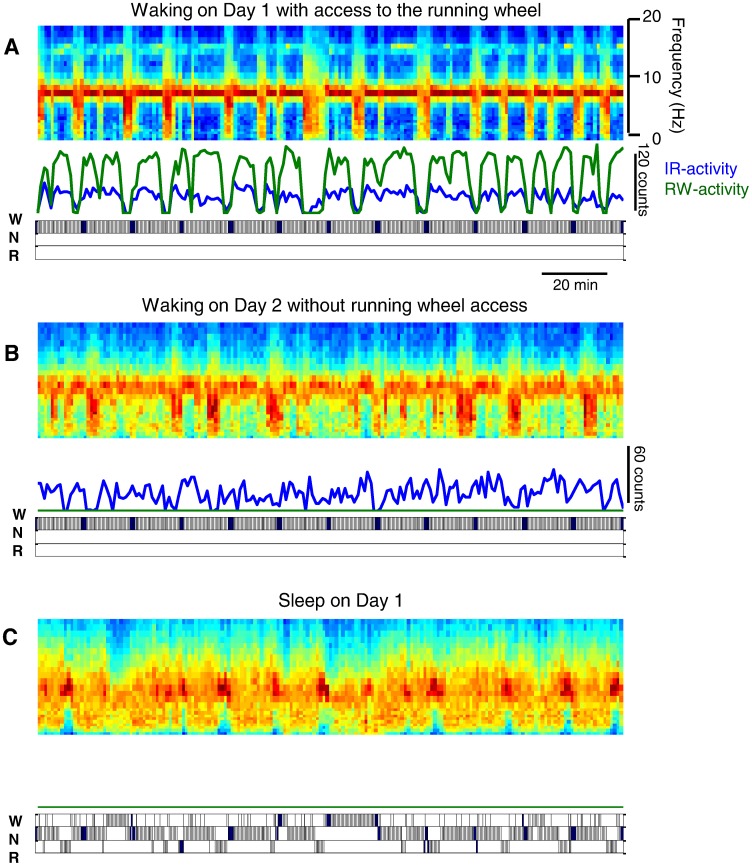
Temporal structure of behaviour and brain activity. A periodic structure is manifested on all three days: in brain EEG activity on the day with access to the running wheel (**A**), during waking without access to the running wheel on Day 2 (**B**) and during sleep on Day 1 (**C**). Each panel depicts a 3-h interval (panels **A** and **B**: beginning 1.5 hours after dark onset on Day 1 and 2 respectively; panel **C**: 5 h after light onset on Day 1), for which the spectrogram of the occipital EEG was computed (top in each panel), along with the IR- and RW-activity (middle in each panel). The three vigilance states, waking (W), NREM sleep (N) and REM sleep (R) are shown in the hypnograms below each panel. Note that the overall amplitude of changes during spontaneous waking on Day 2 was lower than on Days 1 or 3 (the scaling on the color plots in Fig. 5 is adjusted to the maximal values).

Fig. 4 illustrates a typical example of the regular alternation between Run and no-Run bouts, where no-Run bouts are invariably associated with a disappearance of fast theta-waves (∼ 9 Hz), and an emergence of mostly slower activities (<∼7 Hz, Fig. 4A). We were careful to include only continuous wakefulness beginning after dark onset until the start of the first consolidated sleep period (see methods and Fig. 3) in this analysis. Therefore, no sleep occurred during the wake period, which consisted exclusively of alternating Run and no-Run bouts (see the hypnograms below the panels). Notably, while the periodicity was especially apparent on those days when the animals had free access to the running wheel (Day 1 and 3), the periodicity was also evident in most animals during spontaneous waking on Day 2, when wheel-access was prevented (Fig. 4B). Remarkably, even without the running wheel, the mice alternated between active behavior (high theta power) and quiet immobile waking (dominated by slower EEG activity) with a periodicity of approximately 5–20 min. This periodic structure of waking behavior resembled the architecture within sleep, which is characterized by a regular alternation of NREM and REM sleep episodes (Fig. 4C).

**Figure 5:**
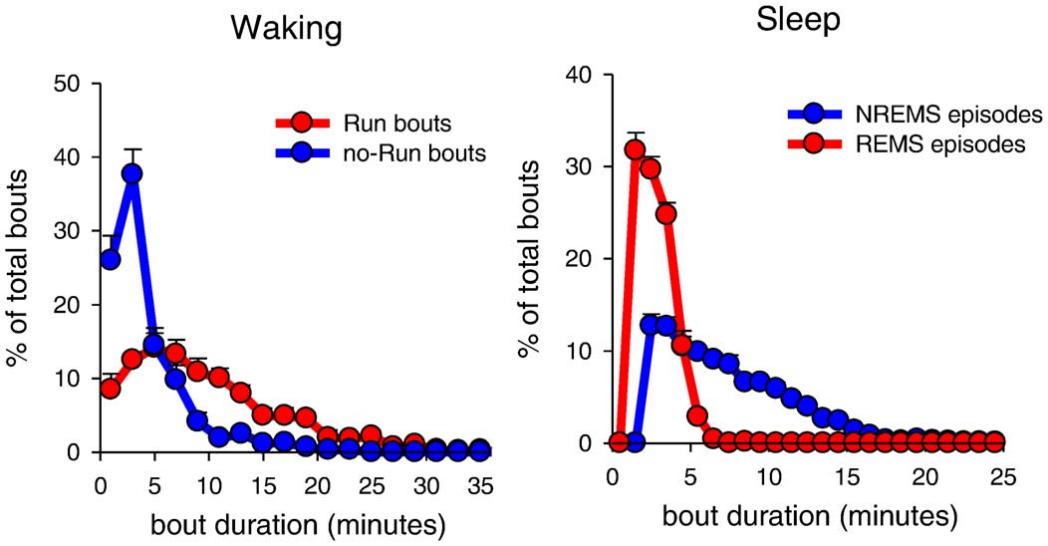
Fine temporal structure of waking and sleep. (**A**) Distribution of all Run and no-Run bouts as a function of their duration and (**B**) of the duration of NREM and REM sleep episodes. Mean values +SEM (n = 8 mice).

Computing the distribution of Run and no-Run bouts according to their duration (Fig. 5A) revealed that Run bouts were on average longer compared to no-Run bouts (Run: 9.7±0.7 min, no-Run: 3.9±0.4 min; the longest bouts were 50 and 23 min for Run and no-Run bouts, respectively). Remarkably, the alternation between these two distinct behavioural states in waking resembled the alternation between the two sleep states – NREM and REM sleep (average episode duration: 7.2±0.2 min and 2.8±0.1 min, respectively (Fig. 5B). The similarity was further emphasized by a consistent ratio between the average duration of a Run/no-Run bout (2.76) and a NREM/REM sleep episode (2.6). The difference between the two ratios was not significant (p = 0.71, paired t-test). These observations suggest that during activated states (Run bouts), there is a tendency to shift into a quiet state (no-Run bouts) at roughly the same rate as the tendency to transition into REM sleep, the brain-activated state, from NREM sleep.

**Figure 6:**
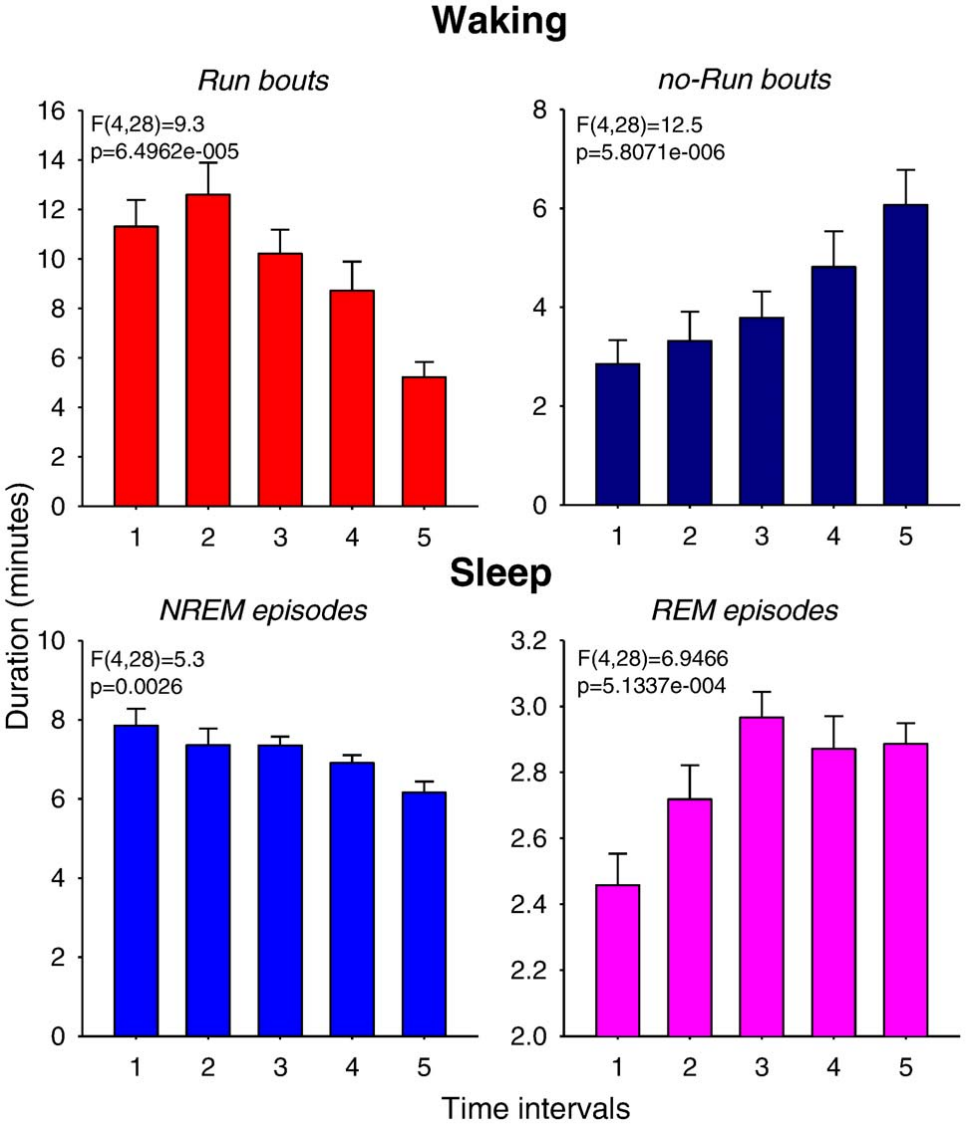
Time course of Run/no-Run bouts and sleep episodes. Time course of the duration of Run and no-Run bouts during waking, and of NREM and REM sleep episodes starting from the first consolidated period of sleep during the dark period until the end of the light phase (all data are means of Days 1 and 3). Since the duration of waking and the timing of sleep onset differed between individuals, the entire time period (waking: Day 1 - 355–677 minutes, Day 3∶404–741 minutes; sleep: the remaining time until the end of 24 h) was subdivided into 5 time intervals, each comprising 1/5th of all waking bouts or sleep episodes. Mean values+SEM (n = 8); one-way ANOVA, factor ‘time’.

Finally, we investigated whether the duration of Run and no-Run bouts and NREM and REM sleep episodes changes systematically across time. For this analysis, we subdivided the waking period, starting from dark onset until the first consolidated sleep episode (Fig. 6, top panels, ‘Waking’), and the remaining time interval from the first consolidated sleep episode (still in the dark period) until the end of 24 h (Fig. 6, bottom panels, ‘Sleep’), into five intervals, each consisting of the same number of bouts or episodes, respectively (see Methods section). All four variables showed highly significant change across time. Specifically, the duration of Run bouts declined progressively after a period of stable duration, while no-Run bout duration increased steadily (Fig. 6, top panels). The observation that the duration of Run bouts was largely stable for several hours at the beginning of the night rules out the possibility that its duration is primarily driven by muscular fatigue. Instead, the major implication of this result is that the duration of Run and no-Run bouts does not seem randomly distributed or merely changing as a function of time of day, but rather reflects the preceding waking history. Consistently, NREM sleep episodes were longest at the beginning of the major sleep period, when sleep pressure is naturally high (Fig. 6, bottom, left). Subsequently, as sleep pressure decreases, NREM sleep episodes shortened progressively, concomitant with a prolongation of REM sleep episodes. To address whether the time of day contributes to these effects, we then subdivided the ‘waking’ period (as defined above) into equal time intervals, rather than based on the number of bouts as above (not shown). The changes in Run and no-Run bout duration across time were again highly significant, despite large differences in sleep latency (up to >5 hours difference between individuals) with respect to the time of day. In other words, at the same time of day within 24 h, some animals were still running and hardly showed any noticeable change in their waking behaviour, while other animals had already progressed through shortening of Run bouts into sleep. This analysis, along with the observation of marked interindividual differences in the total waking duration (see Fig. 1A), suggests that it is unlikely that the dynamics of waking behaviour are driven solely by circadian time. Thus, our results show that both waking and sleep are temporally organized on a short (minutes), and on a long (hours) time scale. Moreover, the two do not seem to be independent. Next, we investigated in greater detail the relationship between the two processes.

### The Fine Structure of Waking and Sleep is Directly Related to the Slow Homeostatic Process

**Figure 7:**
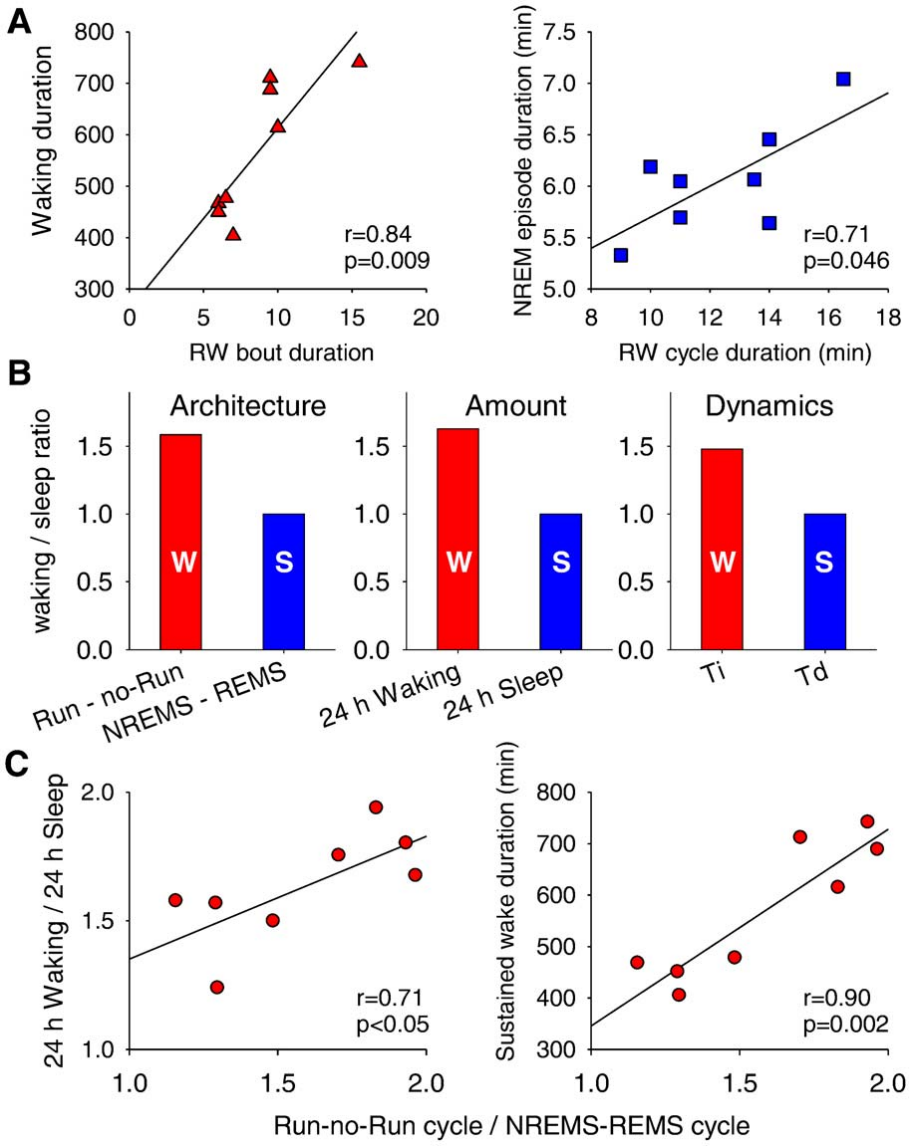
The fine structure of waking and sleep is related to the slow homeostatic process. (**A**) Relationship between the average duration of Run bouts (left) with the duration of waking (starting from dark onset until the occurrence of the first consolidated sleep period), and between the average duration of Run - no-Run cycle and the duration of NREM sleep episodes (right). (**B**) Ratios of average duration of Run - no-Run cycles during waking and NREM - REM sleep cycles (’Architecture’), the total amount of waking and sleep in 24 h (‘Amount’), and the time constants of the Process S, Ti and Td (after Huber et al., 2000; ‘Dynamics’). For all three bar plots wake-related variables (red bars (W): duration of Run - no-Run cycle, total 24-h waking and Ti) are expressed relative to sleep-related variables (blue bars (S): duration of NREM-REM sleep cycle, total 24-h sleep and Td). (**C**) The relationship between the ratio of waking cycle (duration of Run – no-Run cycle) and sleep cycle (duration of NREM – REM sleep cycle) and the ratio of total waking and sleep during 24 h (left) or the capacity to sustain continuous waking (right). R- and P-values resulting from Pearson correlations. Day 3, n = 8 mice for all analyses.

It is still unclear whether sleep propensity in rodents accumulates at a constant rate throughout the waking period, irrespective of ongoing behaviour. Recent studies suggest that the increase in sleep pressure depends on ongoing behaviour [3], [4]. The question remains what ultimately triggers the transition from waking to sleep after sleep pressure reaches the upper threshold of the two-process model of sleep regulation [27], [28]. We hypothesized that the ability to sustain a long waking period is related to the periodicity in the occurrence of Run and no-Run bouts. Indeed, we found that those animals that had shorter Run bouts also terminated the main waking period sooner (Fig. 7A, left). The data suggest that either the homeostatic process accumulates during waking in discrete steps, or that the animals that fall asleep earlier than others tend to show faster transitions from Run- to no-Run state.

Interestingly, the duration of NREM sleep episodes correlated positively with the duration of the Run - no-Run cycle (Fig. 7A, right), or with the duration of Run bouts (r = 0.78, p = 0.02, not shown). We noticed that the average duration of the Run - no-Run cycle (12.4±0.9 min) was longer than the average duration of the NREM-REM sleep cycle (7.8±0.2 min). The ratio between the two cycles (1.58±0.1) appeared to be closely related to the ratio between the total amount of waking and sleep within 24 h (1.63±0.1), the difference between the two being highly non-significant (p = 0.59, paired t-test). It should be noted that the estimated time constants of Process S based on sleep EEG SWA in C57BL/6 mice were also consistently different between waking (Ti = 4.9) and sleep (Td = 3.3) with a ratio of ∼1.5 [27], suggesting that the ratio is common between the total 24-h amount, architecture (bouts and episode duration), and temporal dynamics (time constants of the Process S) of waking and sleep (Fig. 7B).

Intriguingly, the ratios between the 24-h amount of waking and sleep, and the average duration of cycles in waking and sleep were moderately but significantly correlated (Fig. 7C, left), suggesting that those animals which have more consolidated waking, as compared to sleep, also are more awake during 24 h. Moreover, those animals that showed a bias towards longer wake episodes, or a reduced tendency to switch to quiet vs. activated state, appeared to have also a higher capacity for sustaining longer waking periods (Fig. 7C, right). Overall, the data demonstrate that the temporal structure of waking and sleep is a good predictor of the global 24-h sleep-wake behavior, which is regulated homeostatically. These results suggest that the mechanisms triggering the alternations between brain states within waking and sleep may be an integral part of sleep regulatory mechanisms.

## Discussion

The aim of this study was to investigate whether the time scale of the alternation between NREM sleep and REM sleep episodes is relevant for the slow evolution of the homeostatic Process S of the two-process model of sleep regulation. The principal finding was the regular occurrence of shifts between activated and synchronized EEG states throughout sleep (NREM-REM) and, remarkably, also throughout wakefulness (Run - no-Run). We suggest that the natural tendency for state alternations could be an integral part of the “sensing” mechanism, by which the homeostatic process keeps track of the preceding sleep/wake history. The precise temporal organization of both waking and sleep might thus play a crucial role for more efficient sleep timing depending on preceding waking activity. This could, for example, allow an efficient control of the excitability of cortical networks.

We investigated for the first time the fine temporal structure of waking behavior in mice making use of their running wheel activity. We found that running behaviour is organized in distinct bouts, alternating with periods of relative immobility, dominated by slower EEG activity. This tendency to regularly switch from an activated state (Run) to a relatively quiescent (no-Run) state (resembling NREM sleep) during waking appeared to be remarkably similar to the tendency to switch from the synchronized state of NREM sleep to the activated state of REM sleep (resembling waking), during sleep. Such a tendency could indicate that there is an endogenous pressure for a change in brain state, manifested either in the alternation between Run and no-Run bouts, or between NREM and REM sleep episodes. We suggest that the periodicity in waking behaviour may share common substrates, mechanisms, and, ultimately, function with the periodicity in sleep states. One possibility is that such state shifts within waking may serve as “attempts” to fall asleep, while state-shifts during sleep may, in turn, represent the off-line “attempts” to initiate sustained wakefulness.

The specific neurophysiologic mechanisms of state-change propensity remain to be identified. They may be related to the shortage of metabolic substrates, depletion of neurotransmitters, desensitization of synaptic and/or extra synaptic receptors, or a saturation of neuronal circuits. During waking, brief episodes of a relatively immobile quiet state could play a role in the “sensing” mechanism that serves to internally assess the preceding time spent awake. This explanation is plausible, as arousal-promoting neuromodulators, such as norepineprine or orexin, which are released during active waking [40], would likely mask the changes in neuronal and network activity brought about by preceding history. In other words, global brain activity driven by ongoing behavior or sensory inputs (extrinsic) would likely override the local changes in synaptic activity at the level of fine cortical circuits (intrinsic). This mechanism would be particularly relevant for those ionic conductances that are sensitive to the level of membrane depolarization and are affected by arousal-promoting neuromodulators via changes in their voltage-dependent properties [41]. The state of quiet immobility would allow the occurrence of “local sleep” manifested in increased slower EEG activity, likely as a result of relative membrane hyperpolarization [4], [34]. However, if local sleep does not progress into a full-fledged global sleep episode, the animal can stay awake for another cycle, or more, until the upper threshold of the homeostatic process is reached and global sleep can be initiated.

Intriguingly, the duration of individual Run bouts appeared to be a strong predictor of the maximal duration of uninterrupted waking that could be sustained. In other words, the tendency to switch to a different (in this case, quiet) behavioural state was higher if sleep were to occur sooner, even if it was still hours ahead. Importantly, the waking duration that the animals were able to sustain until the first consolidated sleep period was highly variable between individual mice but stable within individuals, suggesting that it is determined by some specific “hard-wired” neurophysiologic substrate. This notion was supported by the observation that the ratio between the duration of Run – no-Run cycles and NREM-REM sleep cycles (∼1.5) was not only similar to the ratio of total 24 h waking and sleep, but the two ratios were correlated. Thus, those animals that have a higher capacity for longer waking also have a stronger tendency to shift to the activated sleep state, REM sleep, also during sleep. Moreover, the ratio between both the 24 h values of waking and sleep, as well as between the waking and sleep cycles, appeared to be remarkably similar to the ratio between the time constants of the increase (Ti) and the decrease (Td) of sleep-wake-dependent process, as estimated previously in the same strain of mice, C57BL/6 (Ti = 4.9, Td = 3.3, ratio = ∼1.5 [27]). We suggest that this similarity has an important implication, as it suggests that the build up of sleep pressure during waking is intimately linked to the tendency to switch between states. As argued above, both the homeostatic increase of sleep need during wake and its dissipation during sleep might occur step-wise in a periodic manner. This would be required for a transient removal (waking) or an enhancement (sleep) of arousal-promoting neuromodulators to unmask the status quo of local and/or global cortical circuits. A precise temporal organization within waking and sleep could therefore play a crucial role in preventing neuronal over-excitability that could compromise waking function, and allow obtaining sleep adequate to the current needs, in terms of duration and intensity.

In conclusion, we found that that the periodicity in waking behaviour may share common substrates, mechanisms and, ultimately, function with the periodicity in sleep states. Our results suggest the intriguing possibility that the short time scale of waking behavior and sleep episodes may be related to the slow homeostatic process.

## Materials and Methods

### Animals

The experiment was approved by the Veterinary Office of the Canton of Zurich. Male C57BL/6 mice (n = 8), 3–4 months old, weight 25–26 g at surgery were used. The mice were kept individually in Macrolon cages (36×20×35 cm), with food and water available *ad libitum*, and maintained on a 12 h light - 12 h dark cycle (daylight type fluorescent tubes, 58 W, approximately 30 lx) at 22–24°C ambient temperature. The animals were provided with a RW (diameter 15 cm, regularly spaced PVC bars; length 5 cm, diameter 3 mm, distance between bars 4 mm: one revolution = 47.1 cm: 1 count). RW- and passive infra-red (IR) activity were recorded continuously throughout a 3-day experiment. RW-counts and IR-activity counts were integrated over consecutive 1-min epochs and stored on a computer as described previously [31].

### Surgery

Epidural electrodes for EEG recording and two wires to record the EMG were implanted under deep anesthesia (ketamin 87 mg/kg - xylazin 13 mg/kg, i.p). Stainless steel electrodes (0–80 X 1/8, Plastics One® Inc., Roanoke, Virginia, USA) were placed over the left and right frontal cortex above the M1 region (2 mm anterior to bregma, 1.5 lateral to the midline; [42]), and over the left and right parietal cortex (3 mm posterior to bregma, 2 mm lateral to midline) and as reference over the cerebellum. The electrodes were connected to stainless steel wires that were fixed to the skull with dental cement. At least 12 days were allowed for recovery after surgery and adaptation to the recording conditions. The mice were provided with a RW for more than 30 days prior to the sleep recordings.

### Experimental Protocol, Data Acquisition and Analysis

The EEG and EMG were recorded with a portable recording system (Institute of Pharmacology and Toxicology, Zurich, Switzerland) for three consecutive days. Before each recording, a calibration signal (10 Hz sine wave, 300 µV peak to peak) was recorded on the EEG and EMG channel. Both signals were amplified (amplification factor approx. 2000), conditioned by analog filters (high-pass filter -3 dB at 0.16 Hz), and sampled with 512 Hz. The signals were filtered by a digital (FIR) filter: EEG: low-pass filter: 0 dB at 30 Hz; EMG: band-pass filter: 0 dB at 20–40 Hz. EEG power spectra were computed for 4-s epochs as described previously [31]. RW activity was analyzed for two days (Day 1 and Day 3, separated by 24 hours, during which access to the RW was prevented (Day 2)). The running bouts (Run) were defined as uninterrupted time periods with more than one RW count per minute in each consecutive 1-min epoch (see Fig. 3A). A no-Run bout was defined as interruption of RW-activity lasting at least 1 min with no more than 1 count/min in each consecutive 1- min epoch (see Fig. 3A). A Run – no-Run cycle was defined as a period starting from the beginning of a Run bout until the end of the immediately following no-Run bout (see Fig. 3A). The duration of Run and no-Run bouts were quantified for the time period starting from dark onset until an occurrence of the first consolidated sleep period (see below, Fig. 1C). During no-Run bouts, the mice were always awake, as manifested by the significantly higher EMG- and IR-activity and lower EEG SWA during no-Run epochs as compared to sleep (see Results section).

To investigate the time course of Run- and no-Run bout duration (Fig. 6, top panels), we used two complementary approaches. In the first approach, we binned the entire waking period from dark onset until the occurrence of the first consolidated sleep period (see below) into five time intervals each consisting of the same number of Run- or no-Run bouts. As a result, intervals were not of the same duration but consisted of the same number of Run- or no-Run bouts. In the second approach, we subdivided the entire waking period into five intervals of identical duration, irrespective of how many Run or no-Run bouts occurred in each of them.

### Vigilance States

The three vigilance states NREM sleep, REM sleep and waking were scored off-line for 4-s epochs by visual inspection of the parietal EEG and EMG records, and EEG power in the slow-wave range (0.75–4.0 Hz). NREM sleep episodes were defined as periods of sleep that lasted for at least 1 min and allowing brief awakenings (consolidated waking interruptions < = 16 s) [43]. A NREM-REM sleep cycle was defined as a period consisting of one NREM sleep episode that was immediately followed by uninterrupted REM sleep, which lasted for at least 32 sec [21]. Shorter REM sleep “attempts” were not included in the analyses. The latency to the first consolidated sleep period was defined as time elapsed between dark onset and the first sleep period that contained at least 5 min of sleep (NREM+REM sleep), whereby interruptions by brief awakenings of < = 16 sec were allowed [31].

To investigate the time course of NREM sleep and REM sleep episode duration (Fig. 6, bottom panels), we used the same strategy as for Run and no-Run bouts (see above), with the exception that the analyses were performed for the time period starting from the first consolidated sleep episode after the dark onset until the end of the light period.

### Statistical Analysis

Comparisons were performed by one-way ANOVA factor ‘time’ and by two-tailed paired t-test (MATLAB, The Math Works, Inc., Natick, MA, USA). The relationship between waking, RW-activity and sleep was assessed by Pearson’s linear correlation analyses.
